# Analysing the Action Research Arm Test (ARAT): a cautionary tale from the RATULS trial

**DOI:** 10.1097/MRR.0000000000000466

**Published:** 2021-03-17

**Authors:** Nina Wilson, Denise Howel, Helen Bosomworth, Lisa Shaw, Helen Rodgers

**Affiliations:** a Biostatistics Research Group; b Stroke Research Group, Population Health Sciences Institute, Newcastle University, Newcastle upon Tyne, UK

**Keywords:** Action Research Arm Test, clinical trial, floor and ceiling effects, stroke

## Abstract

Many studies of stroke rehabilitation use the Action Research Arm Test (ARAT) as an outcome, which measures upper limb function by scoring the ability to complete functional tasks. This report describes an issue encountered when analysing the ARAT subscales in a trial of upper limb therapies after stroke. The subscales of the ARAT at three months followed a ‘U-shaped’ distribution, and therefore, comparing means or medians was not appropriate. A simple alternative approach was chosen that dichotomised the subscales. When analysing the ARAT, the shape of the distributions must be checked in order to choose the most appropriate descriptive and inferential statistical techniques. In particular, if the data follows a ‘U-shaped’ distribution, a simple dichotomising or a more sophisticated approach is needed. These should also be considered for heavily skewed distributions, often arising from substantial floor or ceiling effects. Inappropriate analyses can lead to misleading conclusions.

## Introduction

Many clinical studies of upper limb rehabilitation after stroke use the ARAT as an outcome. It measures upper limb function by scoring the ability of a participant to complete a range of functional tasks [1]. The scale consists of 19 items rated on a four-point ordinal scale ranging from zero (cannot perform any part of task) to three (performs task normally). The overall total has a range of 0–57, but the items can be reported as four subscales (grasp, grip, pinch, gross movement).

The ARAT has generally good psychometric properties [2], but the extent of floor and ceiling effects is still unclear. A floor effect is when many participants obtain the minimum possible score, whereas a ceiling effect is when many participants obtain the maximum score. The existence of these raises doubt whether the scale really covers the full range of ability being measured. A review [2] found that the percentage of participants with the highest or lowest values of the ARAT total score varied considerably across studies, with many reporting percentages above 15%. At this level, lower reliability and responsiveness of the scale are considered [3]. The extent of these effects is likely to vary with the characteristics of the assessed stroke participants, as the distribution of scores shifts, indicating more or less functional limitation. For instance, in the VECTORS study [4], the median ARAT total score was 51.5 (out of 57), whereas, in a study by Hsueh and Hsieh [5], the median ARAT total score was 0: not surprisingly, the first study reported a high ceiling effect (41%), while the second reported a high floor effect (52%).

Although the ARAT can be reported as subscales, not all studies do: the psychometric properties have not been validated for the subscales [6]. There is less evidence on whether floor and ceiling effects occur when using the subscales, but Hsueh and Hsieh [5] reported substantial floor effects on all subscales and some evidence of ceiling effects. The VECTORS study did not report these effects for subscales, but since the median values for grasp, grip and gross movement were the maximum possible, substantial ceiling effects are likely. Besides the consideration of whether the scale covers the full range of abilities, another issue is how to analyse a measurement that potentially has a substantial proportion of data values at the minimum or maximum value.

## Methods, results and discussion

**Fig. 1:**
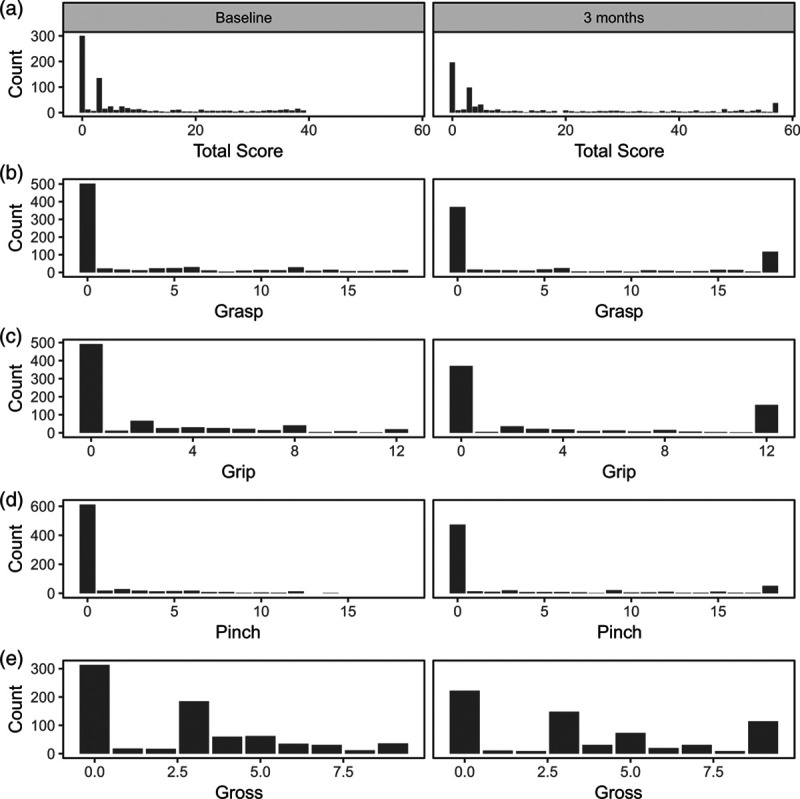
Distribution of the ARAT total score and subscales at baseline (*n* = 769) and 3 months (*n* = 669 except gross where *n* = 668) for RATULS. ARAT, Action Research Arm Test.

The ARAT was reported in the RATULS trial [7]. This compared robot-assisted training with enhanced upper limb therapy and usual care for 770 stroke patients with moderate or severe upper limb functional limitation (baseline ARAT total <39). The primary outcome was whether a participant had achieved an improvement over time of a given size in the ARAT total, but secondary outcomes included the total and ARAT subscales. The median ARAT total was 3 at baseline, so this patient group started with predominantly low scores and, therefore, considerable arm function limitations. The distribution of the ARAT total at baseline and three months shows substantial floor effects (Fig. 1a). Given this feature, we considered how best to compare scores between randomisation groups: this included both descriptive statistics and inferential approaches. Since we wished to adjust any comparison at three months for time since stroke, study centre and baseline ARAT total, some form of multivariate regression was necessary. The analysis could have used either linear regression comparing means or quantile regression comparing medians. In our case, the distribution of the ARAT total was clearly positively skewed at both time points, so comparing means might not seem the obvious approach. However, the requirements for the use of multiple regression techniques look at the shape of the distribution after adjustment for baseline values. This produced normal errors when comparing means at three months after adjustment, and therefore, this was appropriate for the analysis of the total score.

Where ARAT subscales were reported, they have usually been summarised as either a mean [4, 8–11] or median [5,12]: ANOVA and Kruskal–Wallis tests have been used but the distribution shapes that led to these choices were not mentioned. A statistical analysis plan must consider the shape of the data distribution to make appropriate choices. The distribution of the ARAT subscales at three months in RATULS were ‘U-shaped’ rather than the positive skew seen in the total score (Fig. 1b–e), meaning that participants tended to score zero or full marks on each subscale, and few scored the values in-between. This is shown by the substantial floor, and to a lesser extent, ceiling effects (Table 1). Therefore comparing means or medians was not appropriate: neither measure gives a typical value. After consideration of analysis options, we chose to use a simple approach by dichotomising the subscales to give a binary measure and then using logistic regression to compare groups. The split was chosen to be between participants who could complete at least one task of the subscale (scored 2 or 3 on at least one item of that subscale, indicating they completed the task but possibly taking a very long time) and those that could not (scored 0 or 1 on all items of the subscale, indicating that there was no movement or just a partial performance of the task) [13]. More sophisticated analysis techniques could have been chosen [14,15], but these would have made interpretation of the results harder for nonstatisticians.

**Table 1:** Floor and ceiling effects for RATULS and BOTULS at baseline and 3 months

	RATULS		BOTULS	
Outcome*n*	Baseline769	3 months669	Baseline332	3 months312
Grasp (0–18), *n* (%)				
Floor effect	502 (65%)	370 (55%)	212 (64%)	185 (59%)
Ceiling effect	13 (2%)	117 (17%)	5 (2%)	13 (4%)
Grip (0–12), *n* (%)				
Floor effect	492 (64%)	371 (55%)	215 (65%)	193 (62%)
Ceiling effect	20 (3%)	155 (23%)	12 (4%)	19 (6%)
Pinch (0–18), *n* (%)				
Floor effect	612 (80%)	473 (71%)	258 (78%)	222 (71%)
Ceiling effect	0 (0%)	51 (8%)	3 (1%)	7 (2%)
Gross (0–9), *n* (%)				
Floor effect	313 (41%)	222 (33%)^a^	73 (22%)	51 (16%)
Ceiling effect	36 (5%)	114 (17%)^a^	11 (3%)	17 (5%)

a *n* = 668 due to missing data.

**Fig. 2:**
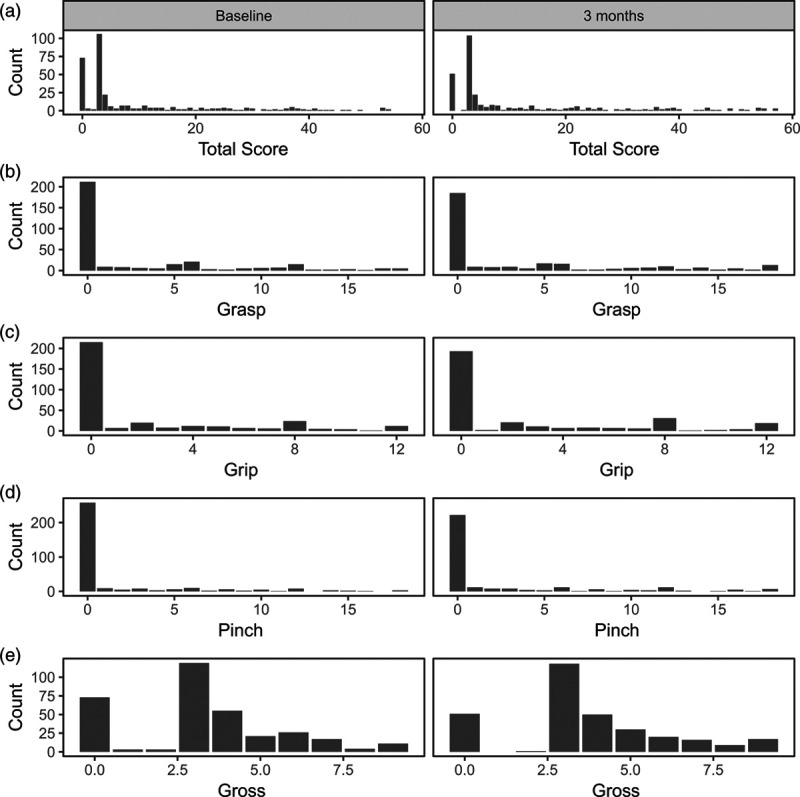
Distribution of the ARAT total score and subscales at baseline (*n* = 332) and 3 months (*n* = 312) for BOTULS. ARAT, Action Research Arm Test.

Although floor or ceiling effects have been reported, other studies have not reported a ‘U-shaped’ distribution of the ARAT subscales with floor and ceiling effects present simultaneously, so we looked at the distribution of the ARAT subscale in another trial of 333 patients evaluating treatment of upper limb spasticity due to stroke with botulinum toxin type A (BOTULS) [16] (Fig. 2, Table 1). In BOTULS, participants also exhibited a considerable lack of arm function at baseline (median ARAT total score = 3). The distributions of the subscales were not ‘“U shaped’ at 3 months, but the distributions were problematic, as they were highly positively skewed with a median of zero for three subscales (i.e. a substantial floor effect). Comparisons of either means or medians across subgroups would be problematic, so a similar approach dichotomising the subscales, as used in RATULS, would be more appropriate.

### Conclusion

When analysing the ARAT total and subscales, care must be taken to check the shape of the data distributions and choose the most appropriate descriptive and inferential statistical techniques. If the data has a ‘U-shaped’ distribution, an alternative to the estimation of means or medians is needed. This should also be considered for heavily skewed distributions, which may result from substantial floor or ceiling effects. Inappropriate analyses can lead to misleading conclusions.

## Acknowledgements

The views and opinions expressed here are those of the authors and do not necessarily reflect those of the HTA programme, NIHR, the UK National Health Service (NHS), or UK Department of Health. We would like to thank participants, local investigators and site staff, co-ordinating centre staff and members of the trial oversight committees of RATULS and BoTULS for their contribution to these research projects.

The employing institutions of all authors received funds from National Institutes of Health Research (NIHR) Health Technology Assessment Programme (HTA) in order for the main RATULS and BoTULS trials to be undertaken. The RATULS and BoTULS trial results are previously published, and part of this manuscript has been presented as a poster at the European Stroke Organisation Conference in May 2019.

## Conflicts of interest

There are no conflicts of interest.
